# Integrated analysis of the functions and clinical implications of exosome circRNAs in colorectal cancer

**DOI:** 10.3389/fimmu.2022.919014

**Published:** 2022-07-18

**Authors:** Tianxiang Lei, Yongxin Zhang, Xiaofeng Wang, Wenwei Liu, Wei Feng, Wu Song

**Affiliations:** ^1^ Department of Gastrointestinal Surgery, The First Affiliated Hospital, Sun Yat-sen University, Guangzhou, China; ^2^ Laboratory of General Surgery, The First Affiliated Hospital, Sun Yat-sen University, Guangzhou, China; ^3^ Center for Digestive Disease, The Seventh Affiliated Hospital of Sun Yat-sen University, Shenzhen, China

**Keywords:** exosome, circRNA, colorectal cancer, diagnostic model, competing endogenous RNA network, immune-infiltrating cell

## Abstract

**Background:**

Exosome circRNAs (Exo-circRNAs) regulate cancer progression and intercellular crosstalk in the tumor microenvironment. However, their biological functions and potential clinical importance in colorectal cancer (CRC) remain unknown.

**Methods:**

We used exoRBase 2.0 data to identify significant differentially expressed Exo-circRNAs (Exo-DEcircRNAs) in CRC patients and healthy individuals. The least absolute shrinkage and selector operation algorithm, support vector machine-recursive feature elimination, and multivariate Cox regression analyses were used to select candidate Exo-circRNAs and constructed a diagnostic model. Quantitative reverse transcription-polymerase chain reaction analysis was performed to confirm the expression of Exo-circRNAs in the serum samples of patients. Furthermore, we constructed an exosome circRNA-miRNA-mRNA network for CRC. Upregulated target mRNAs in the exosome competing endogenous RNA (Exo-ceRNA) network were used for functional and pathway enrichment analyses. We identified 22 immune cell types in CRC patients using CIBERSORT. Correlation analysis revealed the relationship between Exo-ceRNA networks and immune-infiltrating cells. The relationship between target mRNAs and immunotherapeutic response was also explored. Finally, using the Kaplan–Meier survival curve, a prognostic upregulated target mRNA was screened. We constructed a survival-related Exo-ceRNA subnetwork and explored the correlation between the Exo-ceRNA subnetwork and immune-infiltrating cells.

**Results:**

The constructed diagnostic model had a high area under the curve (AUC) value in both the training and validation sets (AUC = 0.744 and AUC = 0.741, respectively). qRT-PCR confirmed that the Exo-circRNAs were differentially expressed in CRC serum samples. We constructed Exo-ceRNA networks based on the interactions among seven upregulated Exo-DEcircRNAs, eight differentially expressed miRNAs, and twenty-two differentially expressed mRNAs in CRC. Functional enrichment analysis revealed that the upregulated target mRNAs were significantly enriched in cytoskeletal motor activity and the PI3K-Akt signaling pathway. Co-expression analysis showed a significant correlation between the Exo-ceRNA networks and immune cells. The significant correlation was observed between target mRNAs and the immunotherapeutic response. Additionally, based on the prognostic upregulated target gene (RGS2), we constructed a survival-related Exo-ceRNA subnetwork (Exosome hsa_circ_0050334-hsa_miR_182_5p-RGS2). CIBERSORT results revealed that the Exo-ceRNA subnetwork correlated with M2 macrophages (*P* = 4.6e-07, R = 0.31).

**Conclusions:**

Our study identified an Exo-diagnostic model, established Exo-ceRNA networks, and explored the correlation between Exo-ceRNA networks and immune cell infiltration in CRC. These findings elucidated the biological functions of Exo-circRNAs and their potential clinical implications.

## Introduction

Although the combination of surgery, chemotherapy, targeted therapy, and immunotherapy has partially improved the clinical efficacy of colorectal cancer (CRC) therapy, CRC remains the third most common cause of cancer mortality worldwide, with more than 1.85 million cases and 850 000 deaths annually (1, 2). CRC is sporadic in 90% of patients and most CRC cases develop from a preclinical benign precursor, adenoma to invasive cancer over the span of years (3). So, CRC survival is highly dependent on early diagnosis (4).

Circular RNAs (circRNAs) are highly conserved and stable because of their covalently closed structure and high tolerance to exonucleases; these are useful characteristics of potential biomarkers for cancer diagnosis (5, 6). Exosomes are non-invasive biomarkers for the early diagnosis of cancers and have attracted increased attention from researchers (7, 8). Exosomes carry circRNAs, messenger RNAs (mRNAs), microRNAs (miRNAs), long noncoding RNAs (lncRNAs), DNA, lipids, and proteins; these can influence the surrounding microenvironment in both biological and pathological conditions, making them potential liquid biopsy-based biomarkers for the early diagnosis of cancers (9–11). Although all cell types can generate exosomes, tumors cells are especially prone to releasing an excessive amount of exosomes. The level of exosomes in the serum or other body fluids of cancer patients is higher than that in normal donors (12). Various circRNAs have been identified in human serum exosomes (13). These serum exosome circRNAs (Exo-circRNAs) are promising novel biomarkers for clinical detection of cancer (9, 14). Therefore, serum Exo-circRNAs represent a new class of exosome-based cancer biomarkers, with the potential for use in liquid biopsy.

circRNAs mainly function as competing endogenous non-coding RNAs (ceRNA) (15, 16). ceRNA binds to complementary miRNAs, acting as a miRNA sponge to inhibit their function, which results in gene expression and consequent regulation of mRNA expression (17). Exo circRNA-miRNA-mRNA networks influence the development, progression, and invasion of cancers (13, 18, 19). The tumor microenvironment (TME), encompassing both cellular and non-cellular milieus, affects tumor development, progression, and metastasis (20). Further, ceRNA networks regulate the crosstalk between tumor cells and immune cells (21, 22). Therefore, we hypothesize that Exo-circRNAs may function as tumor oncogenes, contributing to the development and progression of CRC. However, the biological functions and potential clinical applications of cancer-derived Exo-circRNAs in CRC remain largely unexplored.

In this study, we aimed to explore the potential clinical applications and biological functions of Exo-circRNAs in CRC using comprehensive bioinformatics analyses. First, we established a 6-Exo-circRNA diagnosis model in the exoRBase 2.0 training cohort using a machine learning algorithm, and we validated the model in the GEO cohort. We then determined the presence of the differentially expressed Exo-circRNAs (Exo-DEcircRNAs) of the diagnostic model in the serum of patients. Second, we constructed a dynamic Exo-ceRNA network based on the Exo-DEcircRNAs and identified 22 mRNAs, which were upregulated through the Exo-circRNA and ceRNA mechanism in CRC. Functional annotations and immune infiltration correlations were analyzed for the 22 upregulated mRNAs. The relationship between 22 upregulated mRNAs and immunotherapeutic response was also explored. Furthermore, we identified a survival-related Exo-ceRNA subnetwork (Exo hsa_circ_0050334-hsa_miR_182_5p-RGS2) and determined the correlation between RGS2 expression and immune cell infiltration in CRC.

## Methods

### Data acquisition and analysis

**Figure 1:**
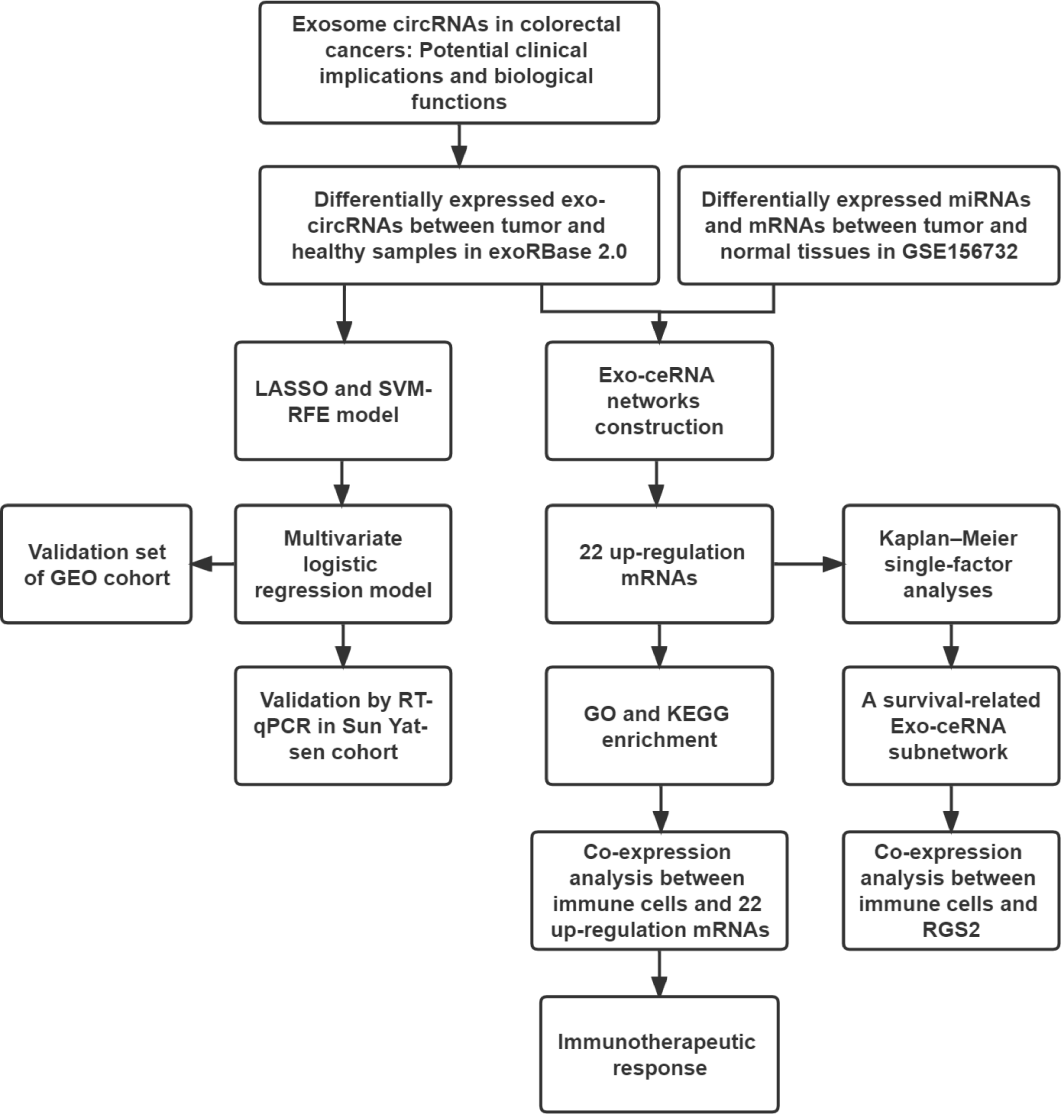
The workflow implemented in this study.

The flowchart of the study is presented in **Figure 1**. To identify differentially expressed Exo-circRNAs (Exo-DEcircRNAs) and explore the potential clinical applications, we downloaded the Exo-circRNA expression profiles from the exoRBase 2.0 database (http://www.exorbase.org/). The dataset included 35 CRC and 118 healthy samples. To construct Exo-ceRNA networks, we also downloaded the dataset GSE156732 from the Gene Expression Omnibus (GEO) database, including mRNA and miRNA expression profiles (three CRC samples and three healthy samples, respectively). In the databases, the differential expressions were determined using the following criteria: |log2 fold change| > 1 and *P*-value <0.01. In addition, RNA profiles (COAD and READ) and survival data (vital status, days to death, and days to last follow-up) were downloaded from the Cancer Genome Atlas (TCGA) database (https://portal.gdc.cancer.gov/). In addition, we downloaded the Exo-circRNA expression profiles from the GSE100063 and GSE100206 as validation cohorts for the diagnostic model. For the therapeutic cohort, three cohorts were employed in the study: IMvigor210 cohort (advanced urothelial cancer with atezolizumab intervention) (23), GSE67501 (renal cell carcinoma with nivolumab treatment) and GSE78220 (metastatic melanoma with pembrolizumab treatment). The information of databases is displayed in the **Supplementary Table 1**.

### Construction and validation of the diagnostic model based on Exo-circRNA

The exoRBase 2.0 database was selected as the training set. First, the least absolute shrinkage and selector operation (LASSO) algorithm and support vector machine-recursive feature elimination (SVM-RFE) were used to filter Exo-DEcircRNAs. We then combined the LASSO and SVM-RFE methods to obtain common Exo-DEcircRNAs. Next, a diagnostic model was constructed using a multivariate logistic regression analysis. Finally, analysis of the receiver operating characteristic (ROC) curve and the area under the curve (AUC) was used to estimate the diagnostic value of the model. The GSE100063 and GSE100206 were selected as the validation sets. To validate whether the diagnostic model had an important diagnostic value, we computed the ROC curve and AUC values of the validation dataset.

### Collection of clinical serum specimens and isolation of exosomes

Twenty-four CRC and twenty-four benign disease serum samples were collected at the First Affiliated Hospital of Sun Yat-sen University from December 2021 to January 2022. The samples were stored at -80°C before exosome extraction. Exosomes were collected from the sera by differential centrifugation. In addition, exosome morphology was identified by transmission electron microscopy (TEM) and nanoparticle tracking analysis (NTA). The expression levels of exosome surface markers, CD9 and HSP70, were evaluated by western blotting. This study was approved by the Ethics Committee of the First Affiliated Hospital of Sun Yat-sen University [approval number (2021):687].

### RNA isolation and quantitative real-time PCR

Total RNA from serum exosomes was extracted using TRIzol reagent (Thermo Fisher Scientific, Waltham, MA, USA) and reverse-transcribed using the PrimeScript RT kit (Takara, China). Quantitative real-time PCR was performed using a SYBR Premix Ex Taq II kit (Takara, China). GAPDH was used as the normalized control for Exo-circRNAs. The relative expression levels of Exo-circRNAs were calculated using the 2^–△△Ct^ method. Primer sequences used in this study are listed in **Supplementary Table 2**.

### Exo-ceRNA network construction

First, we used the cancer-specific circRNA (CSCD, http://gb.whu.edu.cn/CSCD/) to predict the miRNA response elements (MREs) of the Exo-DEcircRNAs. The structural diagram of potential Exo-DEcircRNAs was constructed using the web tool CSCD. Subsequently, we explored differentially expressed miRNAs and mRNAs between CRC and healthy samples using GSE156732. We then identified the miRNAs in the intersection between the MREs of the Exo-DEcircRNAs and differentially expressed miRNAs. We used miRDB (http://mirdb.org), TargetScan (http://www.targetscan.org/vert_72/), and miRTarbase (http://miRTarBase.cuhk.edu.cn/) to predict the target mRNAs of the intersection miRNAs. Furthermore, another intersection was performed between the target mRNAs and differentially expressed mRNAs. Finally, we established an Exo-ceRNA network by matching the Exo circRNA–miRNA and miRNA–mRNA pairs, and the network was visualized using Cytoscape software (Version 3.8.2).

### Functional and pathway enrichment analyses

To further clarify the major functions of the upregulated Exo-circRNAs target mRNAs, we performed Gene Ontology (GO) and Kyoto Encyclopedia of Genes and Genomes (KEGG) pathway analyses. The GO terms and KEGG pathways with *P*-value <0.05 were considered significantly enriched.

### Survival analysis and Exo-ceRNA subnetwork construction

To determine the prognostic characteristics of Exo-ceRNA networks, by combining RNA profiles and survival data from TCGA, we plotted survival curves of the samples with the upregulated target mRNAs based on Kaplan–Meier curve analysis. *P*-values < 0.05 were regarded as significant. The Exo circRNA-miRNA-mRNA subnetwork was constructed based on the prognostic mRNA.

### Prediction of immunotherapeutic response

As mentioned above, three relevant immunotherapeutic cohorts (IMvigor210, GSE67501 and GSE78220) were included and analyzed. In this study, patients who achieved complete response or partial response were categorized as responders and compared to non-responders, who showed signs of progressive disease or stable disease. The Wilcoxon test was used to identify differences in target mRNAs expression between the responder and non-responder groups.

### Comprehensive correlation analysis in tumor-infiltrating immune cells

We used the Cell Type Identification by Estimating Relative Subsets of RNA Transcripts (CIBERSORT) algorithm to estimate the proportions of the 22 immune cell types in the TCGA database (CRC and normal tissues). Only samples with a CIBERSORT output *P*-Value < 0.05 could be subjected to further analysis. The Wilcoxon rank-sum test was performed to determine whether the difference in immune cell proportions between CRC and normal tissues was statistically significant. Furthermore, correlations between the levels of different subsets of infiltrating immune cells and the upregulated target mRNAs were determined and plotted.

## Results

### Construction of a diagnostic model using Exo-DEcircRNAs

**Figure 2:**
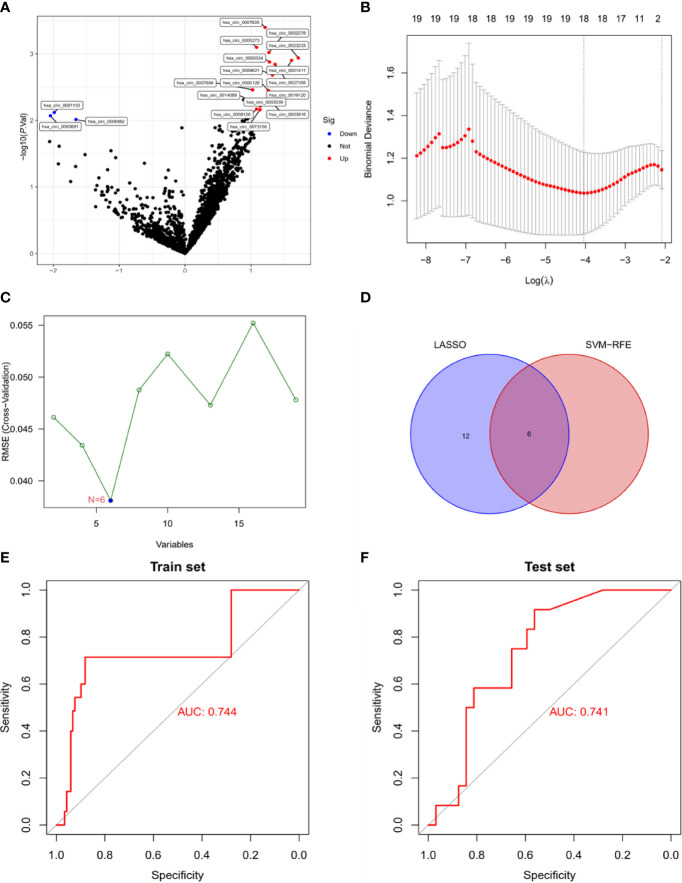
Construction of a diagnostic model using Exo-DEcircRNAs. **(A)** Exo-DEcircRNAs between CRC patients and controls in the exoRBase 2.0 cohort. Blue nodes represent downregulation in CRC; Red nodes, upregulation; and black nodes, no significant difference from controls. **(B)** The 18 diagnostic Exo-circRNAs identified by the LASSO method. **(C)** The six diagnostic Exo-circRNAs identified by the SVM-RFE method. **(D)** The intersection of the diagnostic Exo-circRNAs from the two methods. **(E)** Diagnostic model ROC curves from the training set of the exoRBase 2.0 cohort. **(F)** Diagnostic model ROC curves from the validation set of the GEO cohort. DEcircRNAs, differentially expressed circRNAs; CRC, colorectal cancer; LASSO, least absolute shrinkage and selection operator; SVM-RFE, support vector machine recursive feature elimination; ROC, receiver operating characteristic.

To explore the clinical diagnostic value of Exo-circRNAs in CRC, we used Exo-DEcircRNAs to construct a diagnostic model. RNA sequences of Exo-circRNAs for CRC and healthy samples were downloaded from the exoRBase 2.0 and GEO databases. The training cohort had 19 Exo-DEcircRNAs from the exoRBase 2.0 database (**Figure 2A**; **Supplementary Table 3**). To further narrow down the potential diagnostic Exo-DecircRNAs and construct a diagnostic model, we combined the LASSO and SVM-RFE methods to obtain six common Exo-DEcircRNAs (**Figures 2B–D**). The six final Exo-DEcircRNAs (hsa_circ_0023233, hsa_circ_0001411, hsa_circ_0019120, hsa_circ_0091103, hsa_circ_0063681, and hsa_circ_0006882) formed a diagnostic model using multivariate logistic regression analysis. The formula was as follows: risk score = (expression level of hsa_circ_0023233 × 0.2552) + (expression level of hsa_circ_0001411 × 0.2867) + (expression level of hsa_circ_0019120 × 0.0997) + (expression level of hsa_circ_0091103 × -0.1704) + (expression level of hsa_circ_0063681 × -0.1804) + (expression level of hsa_circ_0006882 × -0.1141).

ROC curve analysis showed that the diagnostic model enabled a more reliable classification between patients with CRC and healthy individuals. The AUC of the diagnostic model was 0.744 for the training set (**Figure 2E**). We used external datasets (GSE100063 and GSE100206) to validate the diagnostic model, and its AUC was 0.741 (**Figure 2F**). These results suggest that the model can effectively distinguish CRC samples from healthy control samples.

### Identification of exosomes and validation of model Exo-circRNAs expression levels

To validate the expression levels of the model Exo-circRNAs, we extracted exosomes from 24 CRC patients and 24 benign patient serum samples. The purity of the exosomes was determined using TEM, NTA, and western blotting (**Supplementary Figures 1A–C**).

**Figure 3:**
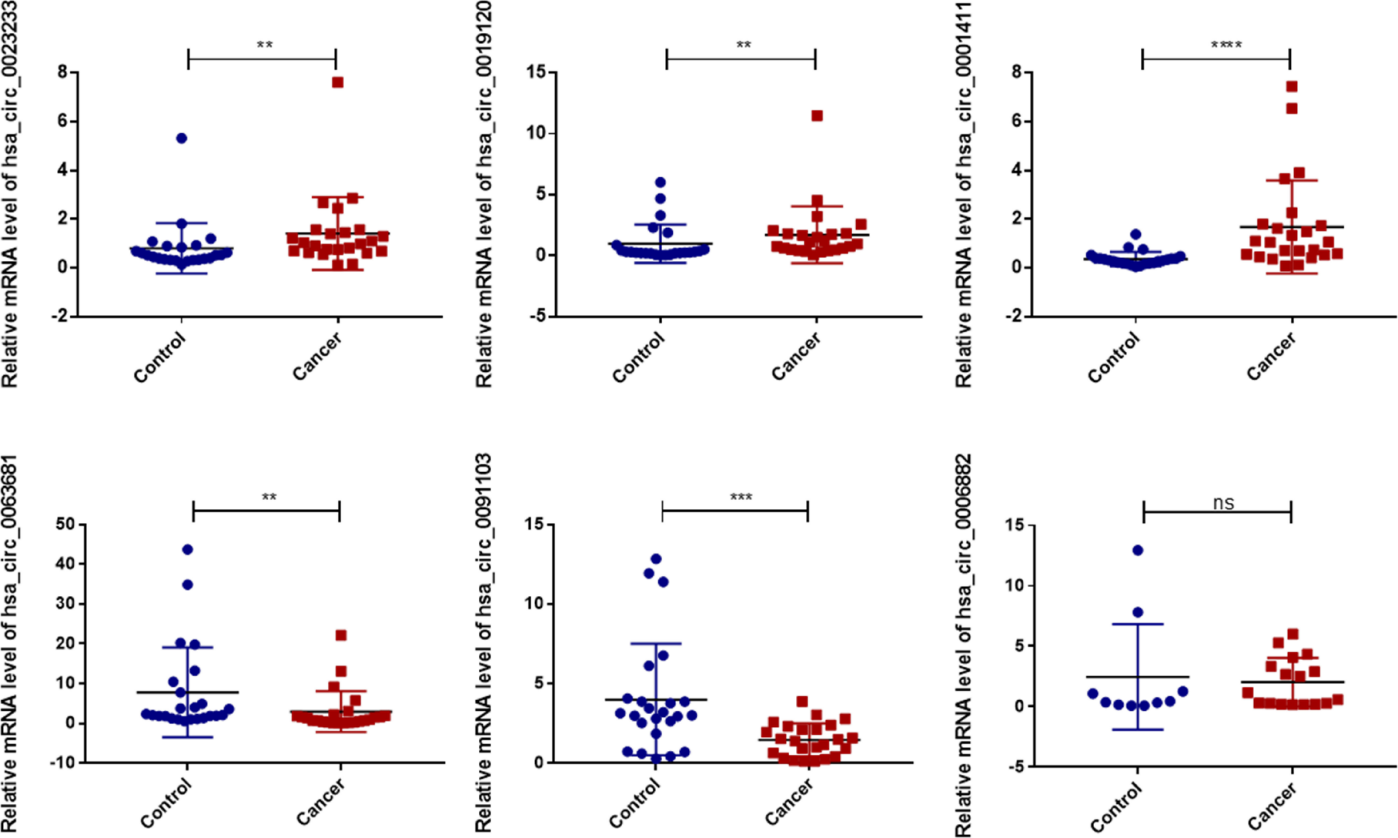
Validation of the diagnostic model Exo-circRNA expression levels in CRC patient serums (n=24) and benign diseases patient serums (n=24). ns, no significance. ****P<0.0001; ***P<0.001; **P<0.01; CRC, colorectal cancer.

We validated the expression levels of the six Exo-circRNAs in the sera of patients with CRC and benign disease (**Figure 3**). The results showed that, in CRC exosomes, the expression levels of hsa_circ_0001411, hsa_circ_0019120, and hsa_circ_0023233 were elevated, whereas hsa_circ_0091103 and hsa_circ_0063681 were downregulated. These results were consistent with the results of the exoRBase 2.0 and GEO profiles.

### Construction and analysis of Exo-ceRNA network

**Figure 4:**
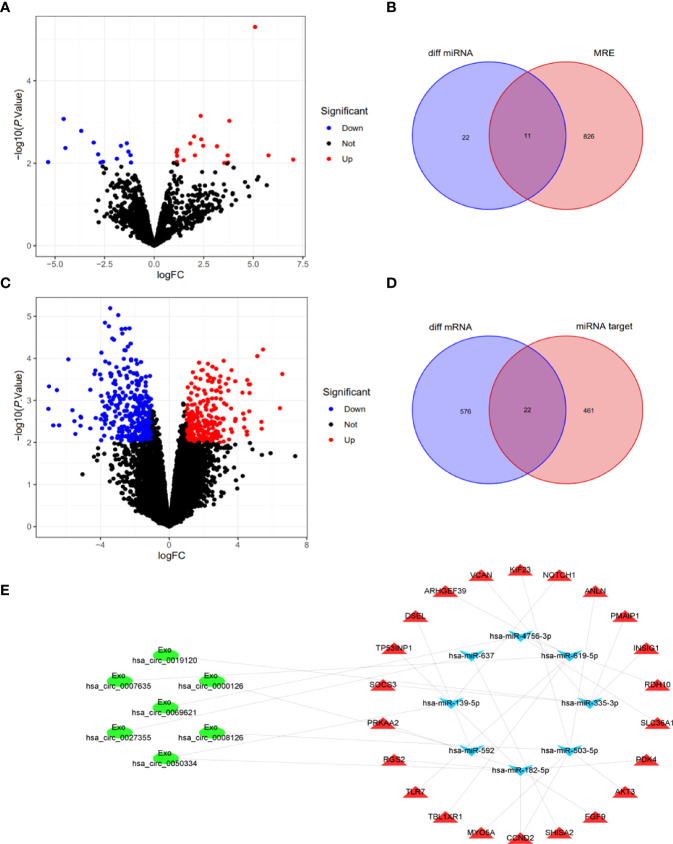
Construction and analysis of Exo-ceRNA network. **(A)** DEmiRNAs between CRC patients and control individuals in the GEO cohort. Blue nodes represent downregulation in CRC; Red nodes, upregulation; and black nodes, no significant difference from controls. **(B)** The intersection of target miRNAs in the MRE of Exo-DEcircRNAs and DEmiRNA. **(C)** DEmRNAs between CRC patients and controls in the GEO cohort. **(D)** The intersection of target mRNAs in the predicted mRNAs, from the miRDB database, TargetScan database and miRTarbase database, and DEmRNAs. **(E)** The Exo-ceRNA (Exo circRNA-miRNA-mRNA) regulatory network: Exo-circRNA, ovals; downregulation miRNA, inverted triangles; upregulation mRNA, triangles. DEmiRNAs, differentially expressed miRNAs; CRC, colorectal cancer; MRE, miRNA response elements; DEmRNAs, differentially expressed mRNAs.

To elucidate the potential functions of Exo-circRNAs in CRC, we constructed and analyzed an Exo-ceRNA network based on the upregulated Exo-DEcircRNAs. First, to identify MRE and target miRNAs, the Exo-DEcircRNAs were predicted using the CSCD database. The pattern maps of the Exo-DEcircRNAs, MRE, RNA-binding protein, and open reading frame information were obtained from CSCD (**Supplementary Figure 2**). We obtained 33 differentially expressed miRNAs (DEmiRNAs) from GSE156732 (**Figure 4A**; **Supplementary Table 4**). In total, 11 eligible miRNAs were selected from the intersection of MRE and DEmiRNAs, as target miRNAs (**Figure 4B**). From GSE156732, we identified 598 differentially expressed mRNAs (DEmRNAs) (**Figure 4C**, **Supplementary Table 5**). Subsequently, the miRDB, TargetScan, and miRTarbase databases were used to predict the target mRNA of the 11 miRNAs. In total, 22 eligible mRNAs were selected as the final upregulated target genes (mRNAs) from the intersection of the DEmRNAs from the various databases (**Figure 4D**).

After multiple screening steps, seven upregulated Exo-DEcircRNAs, eight downregulated miRNAs, and twenty-two upregulated mRNAs were selected for subsequent investigation. The Exo-ceRNA (Exo circRNA/miRNA/mRNA) network was established after a comprehensive consideration of the Exo circRNA/miRNA and miRNA/mRNA interactions. An overview of the Exo-ceRNA regulation network in CRC is displayed in **Figure 4E**.

### Functional and pathway enrichment analysis

**Figure 5:**
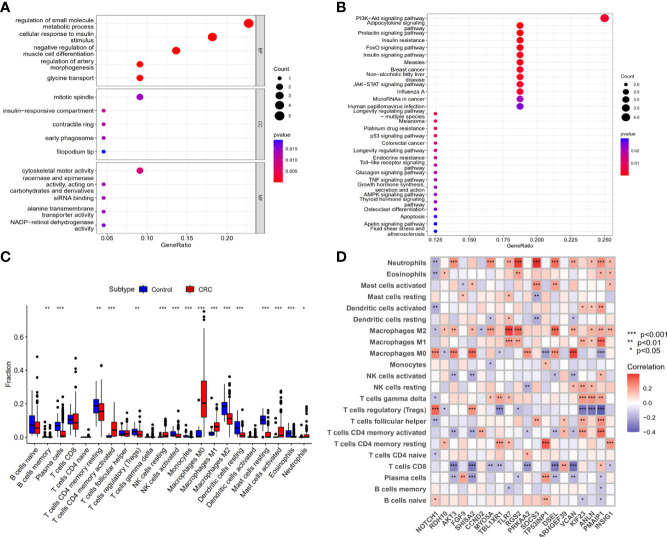
The biological functions of upregulation mRNAs that Exo-circRNAs regulated through the ceRNA mechanism. **(A)** GO enrichment analysis of the upregulation mRNAs. **(B)** The KEGG pathway of the upregulation mRNAs. **(C)** A box diagram of the 22 types of immune cells. **(D)** The correlation between the immune cells and the 22 upregulation target mRNAs. *P<0.05; **P<0.01; ***P<0.001.

We explored the potential function of the downstream genes (mRNAs) that are regulated by Exo-circRNAs *via* the ceRNA mechanism, in CRC. GO and KEGG enrichment analyses were performed. For biological process (BP) enrichment, these genes were mostly enriched in the regulation of small-molecule metabolic processes and cellular responses to insulin stimuli. For cellular component (CC) enrichment, these genes were mainly involved in the mitotic spindle and insulin-responsive compartments. The molecular function (MF) enrichment showed that these genes were mainly enriched in cytoskeletal motor activity and racemase and epimerase activity (acting on carbohydrates and derivatives) (**Figure 5A** **;** **Supplementary Table 6**). KEGG enrichment analysis revealed that these genes were also enriched in the PI3K-Akt and adipocytokine signaling pathways (**Figure 5B** **;** **Supplementary Table 7**).

### Association between the 22 target genes and tumor-infiltrating immune cells in the tumor microenvironment

The relationship between the Exo-ceRNA regulatory axis and immune cell infiltration was investigated. The results indicated that the expression levels of *NOTCH1*, *RDH10*, *AKT3*, *FGF9*, *SHISA2*, *CCND2*, *MYO5A*, *TBL1XR1*, *TLR7*, *RGS2*, *PRKAA2*, *SOCS3*, *TP53INP1*, *DSEL*, *ARHGEF39*, *VCAN*, *KIF23*, *ANLN*, *PMAIP1*, *INSIG1*, *SLC36A1*, and *PDK4* increased stepwise with an increase in Exo-circRNA expression level.

The composition of tumor-infiltrating immune cells was evaluated using the CIBERSORT algorithm, in both the control and CRC samples. Moreover, the association of different types of tumor-infiltrating immune cells in CRC was evaluated (**Supplementary Figure 3**). The results of the Wilcoxon rank-sum test revealed significant differences in the proportions of immune cells between the control and CRC samples (**Figure 5C**). Correlation relationships (Pearson analysis) between immune cells and the 22 target mRNAs were further analyzed and are illustrated in **Figure 5D**. These results suggest that the downstream genes (mRNAs) regulated by Exo-circRNAs *via* the ceRNA mechanism in CRC, are important for immune cell infiltration in tumor pathology.

### Immunotherapeutic response of the 22 target genes

In IMvigor210 cohort, the significant differences in *NOTCH1, KIF23, ANLN, DSEL* and *ARHGEF39* expressions were evident between the responder and non-responder groups (**Supplementary Figure 4**). In GSE78220 cohort, *FGF9* and *VCAN* exhibited a statistically significant difference between two groups (**Supplementary Figure 5**). However, in the GSE67501 cohort, there is no significant difference in expressions of 22 target genes between two groups (**Supplementary Figure 6**).

### Construction of survival-related Exo-ceRNA subnetwork

**Figure 6:**
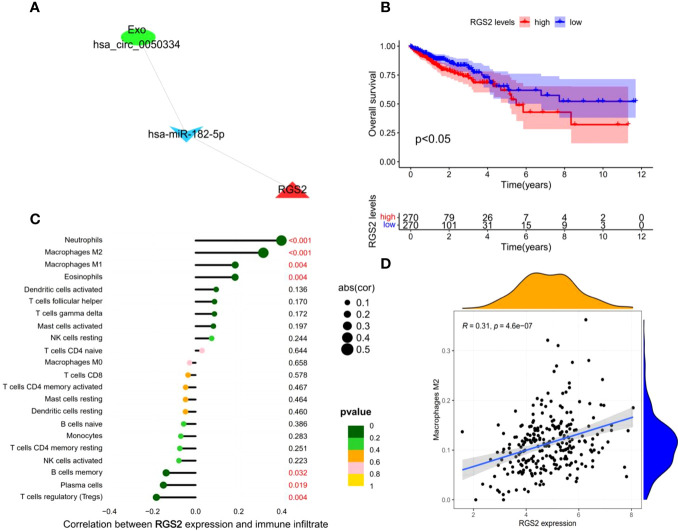
Construction and analysis of survival-related Exo-ceRNA subnetwork. **(A)** survival-related Exo-ceRNA subnetwork. **(B)** TCGA data indicating that the high expression levels of RGS2 are associated with poor prognostic survival. **(C)** The correlation between the immune infiltrating cells and RGS2. **(D)** The correlation between the M2 macrophages and RGS2.

Based on the clinical data of CRC in TCGA, we conducted a Kaplan–Meier single-factor analysis of the 22 target mRNAs in the ceRNA subnetwork. One survival-related target mRNA, *RGS2*, was identified using the log-rank test. Afterwards, a survival-related Exo-ceRNA subnetwork (Exo hsa_circ_0050334-hsa_miR_182_5p-RGS2) was constructed and a Kaplan–Meier survival curve was plotted (**Figures 6A, B**).

### A significant correlation between the survival-related Exo-ceRNA subnetwork and tumor-infiltrating immune cells in tumor microenvironment

To explore the relationship between the survival-related Exo-ceRNA subnetwork (Exo hsa_circ_0050334-hsa_miR_182_5p-RGS2) and tumor-infiltrating immune cells, the CIBERSORT algorithm was used. There was a significant correlation between RGS2 expression level and the proportion of different types of immune cells, including neutrophils (*P* = 8.3e-11, R = 0.4), M2 macrophages (*P* = 4.6e-07, R = 0.31), M1 macrophages (*P* = 0.0038, R = 0.18), eosinophils (*P* = 0.0039, R = 0.18), regulatory T cell cells (Tregs) (*P* = 0.0041, R = -0.18), plasma cells (*P* = 0.019, R = -0.15), and memory B cells (*P* = 0.032, R = -0.14) (**Figures 6C, D**; **Supplementary Figure 7**). Collectively, these results suggest that the survival-related Exo-ceRNA subnetwork (Exo hsa_circ_0050334-hsa_miR_182_5p-RGS2) may participate in immune responses in the TME by affecting immune cells, especially M2 macrophages.

## Discussion

CRC is a highly malignant cancer with a poor prognosis. Patients with CRC have a substantial risk of recurrence and metastasis (24). CircRNAs can be found in exosomes and may also serves as potential novel biomarkers for various diseases (14). Exo-circRNAs can be transmitted into the blood circulation to perform their biological functions through exosomes, and they could serve as a target for liquid biopsy. Exo-circRNA-based liquid biopsy results demonstrate the potential to distinguish patients with cancer from those without cancer (9). Our study analyzed the differences in Exo-circRNA expression between CRC patients and healthy control participants. A further diagnostic Exo-circRNA model was constructed using Exo-DEcircRNAs based on Lasso and SVM-RFE algorithms, which could serve as valuable tool in the diagnosis of CRC.

Although the functions of circRNAs remain largely unknown, the circRNA-miRNA-mRNA axis is known to regulate the development of different types of cancers (25–27). Exo-circRNAs may play a similar role in CRC (28, 29). Based on this concept, we constructed and analyzed an Exo-ceRNA network, which provided a mechanistic understanding of exosome-mediated circRNA communication and elucidated the functions of Exo-circRNA in promoting CRC progression. In this study, we hypothesized that the seven upregulated candidate Exo-circRNAs function as inhibitors of miRNAs, thereby regulating the expression of miRNA target genes and resulting in increased expression levels of the 22 genes in CRC.

GO and KEGG enrichment analyses are performed to further investigate the potential functions of the 22 genes that were upregulated *via* the Exo circRNA-miRNA-mRNA axis in CRC. Functional and pathway enrichment analysis results showed that the 22 upregulated genes were mainly enriched in cytoskeletal motor activity and activation of the PI3K-Akt signaling pathway. Most importantly, “Colorectal cancer” presented among the enrichment terms of KEGG analysis, and this result indicated that the 22 upregulated genes in the Exo-ceRNA network were involved in the progression of CRC. Interestingly, GO analysis revealed that 22 upregulated genes were associated with immune-related pathways, like pro-B cell differentiation, T cell homeostasis and T-helper 17 type immune response. The 22 upregulated genes are likely to be critical factors in the development and progression of CRC and may also affect the host immune response. To understand the effect of infiltrating immune cells, we performed a correlation analysis between the 22 upregulated genes and immune cell types. These cells play important roles in the complex TME and potentially regulate tumor development in CRC (30, 31). Macrophages are the most abundant infiltrating immune cells in the TME (32). Among the macrophages, M2 macrophages are identified as tumor-associated macrophages that play a predominant role in promoting tumor growth and progression (33, 34). Our results revealed that the density of M2 macrophages was positively correlated with the expression levels of *RDH10*, *AKT3*, *SHISA2*, *MYO5A*, *TLR7*, *RGS2*, *DSEL*, *VCAN*, *ANLN*, *PMAIP1*, and *INSIG1*. The correlation of the upregulated genes with the infiltration of M2 macrophages, suggests that Exo-circRNAs regulate the tumor microenvironment *via* Exo-ceRNA networks; thus, promoting tumor growth and progression. Meanwhile, three relevant immunotherapeutic cohorts (IMvigor210, GSE67501 and GSE78220) were analyzed in our study, which elucidate the immunotherapeutic response was associated with target mRNAs.

Considering the potential clinical value of Exo-ceRNA networks in cancer, we constructed a novel survival-related ceRNA subnetwork (Exo has_circ_0050334-has_miR_182_5p-RGS2). Notably, our study indicated that high *RGS2* expression level was significantly associated with poor prognosis in CRC. Upregulation of Exo hsa_circ_0050334 promoted colorectal cancer progression by activating the Exo hsa_circ_0050334-hsa_miR_182_5p-RGS2 axis, leading to the increased expression level of *RGS2*. RGS2, a regulator of G-protein signaling 2, possesses an RGS domain that mediates its GTPase protein-activating activity towards G-protein subunits, which activates phospholipase C intracellularly to exert a biological effect. Aberrant RGS2 expression has been shown in several types of carcinomas and is associated with negative prognosis, suggesting that the regulation and function of RGS2 is reflective of the cancer cell type and tumor microenvironment (35, 36). Additionally, based on the expression level of *RGS2*, we found seven types of tumor-infiltrating immune cells in CRC tissues; a positive correlation was observed with M2 macrophages (*P* = 4.6e-07, R = 0.31). This may explain the correlation between *RGS2* overexpression and poor prognosis in CRC patients. Therefore, the crosstalk between the Exo hsa_circ_0050334-hsa_miR_182_5p-RGS2 axis and tumor-infiltrating immune cells in CRC, provides a potential therapeutic target for future treatments of CRC.

To the best of our knowledge, this is the first study to comprehensively describe the expression of Exo-circRNAs in CRC. However, potential limitations of this study should be considered when interpreting the findings. First, we could not explore the association between the Exo-circRNAs and the prognosis of CRC due to the lack of therapeutic and prognostic information. Second, because the data we analyzed was obtained from public databases, further experimental studies are required to validate our findings.

## Conclusions

The prognosis of patients with CRC and the proportion of immune cell infiltration in the tumor microenvironment are closely related to Exo-circRNAs. This comprehensive bioinformatic analysis of Exo-circRNAs may provide new insights into the mechanisms of CRC development. Moreover, our diagnostic model exhibits compelling clinical value and may be useful in the development of individualized treatment of patients with CRC.

## Data availability statement

The original contributions presented in the study are included in the article/**Supplementary Material**. Further inquiries can be directed to the corresponding author.

## Ethics statement

The studies involving human participants were reviewed and approved by the Ethics Committee of the First Affiliated Hospital of Sun Yat-sen University [approval number: (2021)687]. Written informed consent for participation was not required for this study in accordance with the national legislation and the institutional requirements.

## Author contributions

TL was the lead investigator and contributed to writing the article. WS, YZ and XW were the chief investigator and thesenior author of the article. WL and WF helped with some analysis and interpretation of data. All authors contributed to the article and approved the submitted version.

## Funding

This project was supported by the National Natural Science Foundation of China (grant Number: 81871908). Guangdong Provincial Natural Science Foundation (grant Number: 2018A030313715). Guangzhou Science and technology plan-General Project (grant Number: 201904010036). General projects of Guangdong Provincial Natural Science Foundation (grant Number: 82070529).

## Conflict of interest

The authors declare that the research was conducted in the absence of any commercial or financial relationships that could be construed as a potential conflict of interest.

## Publisher’s note

All claims expressed in this article are solely those of the authors and do not necessarily represent those of their affiliated organizations, or those of the publisher, the editors and the reviewers. Any product that may be evaluated in this article, or claim that may be made by its manufacturer, is not guaranteed or endorsed by the publisher.

## Supplementary material

Supplementary Figure 1 Identification of exosomes in patient serums. **(A)** Morphologies of exosomes in patients which were observed by transmission electron microscopy. **(B)** The size and concentration of exosomes determined by nanoparticle tracking analysis. **(C)** Exosomes analyzed by western blotting, using antibodies against exosome markers (HSP70 and CD9).

Supplementary Figure 2 The basic structure of the differentially expression Exo-circRNAs in colorectal cancers.

Supplementary Figure 3 Composition of immune cells and association of tumor-infiltrating immune cells in CRC and control samples.

Supplementary Figure 4 The correlation between 22 target genes and the immunotherapeutic response in IMvigor210 cohort.

Supplementary Figure 5 The correlation between 22 target genes and the immunotherapeutic response in GSE78220 cohort.

Supplementary Figure 6 The correlation between 22 target genes and the immunotherapeutic response in GSE67501 cohort.

Supplementary Figure 7 The correlation between the immune cells and RGS2.
