# Supplementary material for: Integrated analysis of the functions and clinical implications of exosome circRNAs in colorectal cancer
Source: Front Immunol. 2022 Jul 18;13:919014. doi: 10.3389/fimmu.2022.919014 (PMC9339618; doi:10.3389/fimmu.2022.919014)
Supplement: Supplementary Figure 1 — Identification of exosomes in patient serums. (A) Morphologies of exosomes in patients which were observed by transmission electron microscopy. (B) The size and concentration of exosomes determined by nanoparticle tracking analysis. (C) Exosomes analyzed by western blotting, using antibodies against exosome markers (HSP70 and CD9). [file Table_1.docx]

**Supplementary Table 1 |** The information of databases

| **Dataset** | **Samples** | **Platforms** | **References (doi)** |
| --- | --- | --- | --- |
| exoRBase 2.0 database | 35 colorectal cancer and 118 healthy samples |  | 10.1093/nar/gkab1085 |
| GSE156732 | 3 colorectal cancer and 3 healthy samples | GPL20712 Agilent-070156 Human miRNA  GPL26963 Agilent-085982 Arraystar human mRNA V5 microarray |  |
| GSE100063 | 12 colorectal cancers samples | GPL11154 Illumina HiSeq 2000 (Homo sapiens) | 10.1093/nar/gkx891 |
| GSE100206 | 32 healthy samples | GPL11154 Illumina HiSeq 2000 (Homo sapiens) | 10.1038/cr.2015.82. Epub 2015 Jul 3. |
| IMvigor210 | 68 patients with anti-PD-1 Response (complete response or partial response), and 230 patients with anti-PD-1 No Response (progressive disease or stable disease) |  | 10.1038/nature25501 |
| GSE67501 | 4 patients with anti-PD-1 Response and 4 patients with anti-PD-1 No Response | GPL14951 Illumina HumanHT-12 WG-DASL V4.0 R2 expression beadchip | 10.1158/2326-6066.CIR-16-0072 |
| GSE78220 | 14 patients with anti-PD-1 Response (complete response or partial response), and 12 patients with anti-PD-1 No Response (progressive disease or stable disease) | GPL11154 Illumina HiSeq 2000 (Homo sapiens) | 10.1016/j.cell.2016.02.065. |
