# Supplementary material for: Integrated analysis of the functions and clinical implications of exosome circRNAs in colorectal cancer
Source: Front Immunol. 2022 Jul 18;13:919014. doi: 10.3389/fimmu.2022.919014 (PMC9339618; doi:10.3389/fimmu.2022.919014)
Supplement: Supplementary Figure 2 — The basic structure of the differentially expression Exo-circRNAs in colorectal cancers. [file Table_2.docx]

**Supplementary Table 2 |** The primers of circRNAs and genes.

| **circRNAs and genes** | **Forward primers** | **Reverse primers** |
| --- | --- | --- |
| hsa_circ_0023233 | 5’-GAGGAACACGGTAGATCTAAACATG-3’ | 5’-CCAGTGGAGTCTTGATCGGTAAT-3’ |
| hsa_circ_0001411 | 5’-CAGTATGGGAGTTATGGTACAGCAC-3’ | 5’-TCTTCGGTCTCAGTCACTTTCTGT-3’ |
| hsa_circ_0019120 | 5’-GTCCAAGATGAAAAATCAGGTGTTG-3’ | 5’-CGGAAGTAGGTTCTTGTCTGGTT-3’ |
| hsa_circ_0091103 | 5’-GGATTTTGCCAATCTTCAGAATTGC-3’ | 5’-GACCAAGGTTGCGTAGAATGC -3’ |
| hsa_circ_0063681 | 5’-GGCAAGTGGACAGTGAGTAACC-3’ | 5’-CTTATTAGATTGAGACACAAACCGC-3’ |
| hsa_circ_0006882 | 5’-GGCAAGCGTAATGTTATTGAAGC-3’ | 5’-ACCCATGTTTAGCAATAAATCCCA-3’ |
| GAPDH | 5’-CAAGGTCATCCATGACAACTTTG -3’ | 5’-GGCCATCCACAGTCTTCTGG -3’ |
